# Developing algorithms for healthcare insurers to systematically monitor surgical site infection rates

**DOI:** 10.1186/1471-2288-7-20

**Published:** 2007-06-06

**Authors:** Susan S Huang, James M Livingston, Nigel SB Rawson, Steven Schmaltz, Richard Platt

**Affiliations:** 1 Channing Laboratory, Department of Medicine Brigham and Women's Hospital Boston, MA, USA; 2 Department of Ambulatory Care and Prevention Harvard Medical School and Harvard Pilgrim Healthcare Boston, MA, USA; 3 Center for Health Care Policy and Evaluation Eden Prairie, MN, USA; 4 GlaxoSmithKline Mississauga, ON, Canada; 5 Clinical Innovations Center Humana, Incorporated Louisville, KY, USA; 6 Division of Research Joint Commission on Accreditation of Healthcare Organizations Oakbrook Terrace, Illinois, USA

## Abstract

**Background:**

Claims data provide rapid indicators of SSIs for coronary artery bypass surgery and have been shown to successfully rank hospitals by SSI rates. We now operationalize this method for use by payers without transfer of protected health information, or any insurer data, to external analytic centers.

**Results:**

We performed a descriptive study testing the operationalization of software for payers to routinely assess surgical infection rates among hospitals where enrollees receive cardiac procedures. We developed five SAS programs and a user manual for direct use by health plans and payers. The manual and programs were refined following provision to two national insurers who applied the programs to claims databases, following instructions on data preparation, data validation, analysis, and verification and interpretation of program output.

A final set of programs and user manual successfully guided health plan programmer analysts to apply SSI algorithms to claims databases. Validation steps identified common problems such as incomplete preparation of data, missing data, insufficient sample size, and other issues that might result in program failure. Several user prompts enabled health plans to select time windows, strata such as insurance type, and the threshold number of procedures performed by a hospital before inclusion in regression models assessing relative SSI rates among hospitals. No health plan data was transferred to outside entities.

Programs, on default settings, provided descriptive tables of SSI indicators stratified by hospital, insurer type, SSI indicator (inpatient, outpatient, antibiotic), and six-month period. Regression models provided rankings of hospital SSI indicator rates by quartiles, adjusted for comorbidities. Programs are publicly available without charge.

**Conclusion:**

We describe a free, user-friendly software package that enables payers to routinely assess and identify hospitals with potentially high SSI rates complicating cardiac procedures.

## Background

Surgical site infections (SSIs) are among the most common healthcare-associated infections [1], and they incur substantial morbidity and mortality. Monitoring and feedback of SSI rates has consistently resulted in improvement of SSI rates when combined with other quality improvement activities [2]. Recently, increased emphasis has been placed on SSI rates as a quality indicator for hospitals, and some state legislatures are requiring hospitals to report SSI rates as part of report cards intended for public use in comparing hospitals to one another (Illinois Public Act 93-0563; Missouri HCS/SS/SCS/SB 1279 - Missouri Nosocomial Infection Control Act of 2004).

The ability of hospitals to monitor SSI rates is limited by three factors. First, current surveillance methods are so resource-intensive that many hospitals are able to monitor only selected procedures. Second, current surveillance definitions include subjective components, such as a surgeon's diagnosis of SSI, which create the opportunity for substantial variation in judgment and documentation. Third, a majority of SSIs occur days or weeks after hospital discharge and elude hospital-based tracking systems [3,4]. Attempts to use patient and surgeon self-reporting via questionnaires or phone calls to identify post-discharge infections are both insensitive and resource-intensive [4-7].

These limitations to conventional surveillance for SSI have prompted the development of surveillance methods based upon automated data that are routinely collected during the delivery of, or payment for, healthcare. Previous work by this group has demonstrated the ability of automated hospital-based pharmacy data and discharge diagnoses codes to significantly improve detection of pre-discharge SSIs [8-11].

Beyond pre-discharge assessment of SSIs, health plans and payers possess automated data on the full breadth of claims for hospitalizations, outpatient visits, antibiotic utilization, and diagnosis codes. This enables automated and comprehensive identification of indicators of infection following a specific type of surgery, regardless of whether care is sought at the same hospital at which the procedure was initially performed [16,17]. An additional benefit is that claims data are largely standardized across medical facilities and providers, enabling a potentially universal method for detection of SSI indicators.

We have previously shown that administrative claims data can identify pre- and post-discharge SSIs [12-16]. The purpose of this prior work was not to measure actual SSI rates in individual hospitals, but rather to be able to relatively rank hospitals by SSI rates to identify potential outliers. Claims-based algorithms were designed to be highly sensitive, but less specific in detecting actual SSIs. Thus, claims-based indicator rates were higher than the actual SSI rates, but nevertheless, were successfully able to rank hospitals by quartiles of SSI rates.

In particular, a claims-based algorithm for identifying SSIs after coronary artery bypass graft surgery (CABG) was validated in several health insurers by extensive medical record review [16]. This algorithm was shown to successfully identify post-surgical infections and provide qualitative rankings of hospitals by SSI rates after adjustment for age, sex, and comorbidities. Furthermore, comparisons of hospitals were also possible using merged data from multiple payer sources.

Importantly, prior work evaluating these algorithms necessarily required the release of health insurer data, including protected health information, to an external academic center for analysis and refinement of the algorithms. In a time where protection of identifiable health information is critically important, the utility of such algorithms depends, in part, on their potential to be used at the payer location by payer-specific programmers and analysts. Our goal was to develop user-friendly software for routine serial use by healthcare insurers to assess relative SSI rates at hospitals performing procedures on their enrollees. This software could be used to track a hospital's relative performance over time, provide information to members in need of surgery, and, ultimately, reduce payer costs by preferring hospitals with relatively low SSI rates for a given procedure.

The purpose of this study was not to re-validate the claims-based algorithms for detecting post-CABG SSIs, but rather to demonstrate that these algorithms could be operationalized into a user-friendly software package for use at the payer level without transfer of claims data to an external analysis center. We now describe a method, and provide accompanying computer programs, to allow the payers to routinely use this approach to identify hospitals that may have high rates of SSIs complicating cardiac procedures.

### Implementation

Technical details for preparing data inputs and applying the software to claims data are provided in the online user manual [20]. This manual and the associated software are publicly available without charge.

## Results

### Description of Application Package

We developed an application that accepts inputs in the form of claims data for patients undergoing cardiac procedures and returns aggregated hospital-specific rates of SSI indicators. The application consists of an instruction set, data dictionary, and a series of five SAS programs (SAS Institute, Cary, NC). In brief, a user manual guides health plan analysts through the steps to format common claims codes into data inputs needed for the application package. Analysts extract claims for patients with cardiac procedures identified by ICD9 codes, and create five files using data layouts that are provided: a surgery file, membership file, demographic file, dispensing file, and inpatient/outpatient utilization file. The programs create patient-level analysis files that combine demographic, inpatient, outpatient, and pharmacy dispensing data to identify patients who have an indicator (e.g. diagnosis or procedure or antibiotic dispensing) suggesting a postoperative infection. It then aggregates this information by the hospital at which the procedure was performed, and ranks hospitals according to the fraction of patients with an indicator of infection. The rankings are case-mix adjusted for the patients' age, sex, and comorbidity, assessed by the chronic disease score [18,19]. This study was approved by the institutional review board of Harvard Pilgrim Health Care.

### Patient-level files

The surgery file identifies cardiac procedures based upon ICD9 codes related to invasive cardiac surgery (36.10–36.19, 36.2) and non-invasive cardiac procedures (35.00–35.04, 35.96, 36.01–36.07, 36.09, 35.10–35.14, 35.20–35.28, 35.33, 39.61, 39.66), along with facility, date of procedure, dates of admission and discharge, discharge status, and type of health plan membership (programs select the two most populous groups, e.g. Medicare, commercial, but user override exists) at the time of the procedure. The membership file includes dates of health plan enrollment, and an indicator of whether or not the member had pharmacy coverage. The demographic file includes gender, date of birth, and the type of health plan coverage (e.g. Medicare or commercial). The dispensing file includes the date, amount, and type of all covered medications dispensed in the ambulatory setting for the period of 182 days prior to the procedure date through 30 days afterward. Finally, the utilization file includes all inpatient and outpatient claims occurring from the admit date associated with the procedure of interest to 30 days afterward. All procedure episodes are linked within the five files by the member's identification number.

### Creation of hospital-level performance measures

The application creates hospital-level aggregate data as follows:

Episodes of cardiac procedures are identified and then categorized as having outpatient pharmacy benefits or not. Pharmacy coverage is judged to be in effect for the peri-operative period if a) any dispensings occur during the six months (182 days) preceding the operation, b) any dispensings occur during the 30 days following the operation, or c) membership information indicates post-operative pharmacy coverage. This approach is technically simpler than assessing the individuals' pharmacy benefits coverage status during this 212 day period; in our experience the correlation between prescription dispensings and pharmacy coverage has been sufficient for this purpose. If at least 80% of episodes are performed in members with pharmacy coverage, then default analyses are restricted to those members with pharmacy benefits, thus enabling the addition of outpatient antibiotic prescriptions as an SSI indicator. If fewer than 80% of patients have pharmacy coverage, then outpatient antibiotic exposure is ignored for all patients, and no patients are ignored because of lack of pharmacy coverage. Inclusion of pharmacy benefit information is preferred, in part because it permits partial adjustment for hospitals' case-mix via a chronic disease score based on the six months of pharmacy dispensing prior to the cardiac procedure [18,19].

These cardiac procedure episodes are assigned sex, gender, insurer type (e.g. Medicare versus commercial), and a chronic disease score (Clark TC score) based on member information [18,19]. Each cardiac procedure episode is then evaluated for the occurrence of SSI indicators within 30 days following the procedure [16]. Inpatient SSI indicators are based upon diagnosis or procedure codes suggestive of infection during either the index admission or a hospital re-admission. Outpatient SSI indicators are based upon diagnosis or procedure codes suggestive of infection during either an emergency department visit or an outpatient provider visit. In addition, if available, pharmacy claims for anti-staphylococcal antibiotics within 30 days of the procedure result in an outpatient SSI indicator for antibiotic usage. These SSI indicators are aggregated by type for each hospital that performs the cardiac procedures of interest.

Prior to any aggregation of data or descriptive output, health plan analysts are directed to a series of prompts to define user options. Available user options are found in Table 1.

**Table 1:** User Options

**Option**	**Default**
1. Time window of Interest	None
2. Maximum number of diagnoses in any single claims record	4
3. Maximum number of procedures in a single claims record	5
4. Definition of two most common plan types	None
5. Acceptable membership gap *	45
6. Threshold % of patients with prescription activity to restrict analyses to those with prescription benefits	≥ 80%
7. Threshold number of cardiac procedures during the time window for a hospital's inclusion in multivariate analyses	40
8. Quartile of hospital SSI rates for detailed patient output	4th

* Brief lapses in enrollment during the time window which do not result in exclusion

### Data Integrity

The program runs a series of validation steps to routines to assist payer-based analysts in identifying common problems, such as incomplete or incorrectly formatted input files that either prevent the programs from running or yield incorrect results. For example, steps direct the user to evaluate whether procedure dates span the entire specified date range, whether the number of procedures is similar across the time window, whether any procedure codes fail to identify any procedures, and whether any other variables or strata generate a zero count. Additional guidance is provided in the user guide [20] to help quality assurance analysts determine whether the data are likely to be complete.

### Data Analyses

The programs create summary tables describing the overall number of total, invasive, and non-invasive cardiac procedures and the percentage with at least one SSI indicator by half-year. SSI indicator rates are further stratified by insurer type and type of indicator, e.g. inpatient indicator versus outpatient indicator.

The programs additionally create detailed tables displaying hospital-specific descriptive data (age, gender, chronic disease score) and hospital-specific numbers of total, invasive, and non-invasive cardiac procedures. Hospitals are ranked by their specific SSI indicator rates, and are further grouped into quartiles. Furthermore, SSI indicators are stratified by insurer type, cardiac procedure type (e.g. total, invasive, non-invasive), indicator type (e.g. total, inpatient, emergency department, ambulatory care, antibiotic), and half-year.

A previously validated multivariate logistic regression model [16] assesses the risk of SSI indicators using patient-level inputs including gender, chronic disease score ≥ 4,500, [21] type of insurer plan (e.g. Medicare versus commercial), and hospital in which the cardiac procedure was performed. The hospital covariate is entered as a categorical variable of hospitals performing >40 cardiac procedures across the selected period of interest. The default threshold of 40 procedures was selected to avoid unstable estimates; however, this threshold can be changed by the user. The model assesses the relative odds of infection conferred by a given hospital in comparison to the risk of infection among all patients having procedures in hospitals with unadjusted SSI indicator rates in the lowest quartile. The adjusted odds ratio for each hospital covariate can then be compared against unadjusted rankings.

Among top quartile hospitals with at least 40 procedures, the program provides a line list of procedures that have at least one associated SSI indicator. Individual membership information is provided along with procedure date, procedure location, procedure code and text, and associated SSI indicators by type, date, location, and claims code and text.

### Testing of Application Package

We worked with health plans affiliated with UnitedHealth Group (UHG) [22] and Humana Incorporated to develop and test these programs. After a few iterations involving data validation steps such reformatting of pharmacy inputs, algorithms ran successfully. These attempts enabled us to consider and provide comprehensive data validation routines.

The application package was tested using a slightly earlier version than the one released with this publication. In particular, it involved small differences in ICD-9 codes for both invasive (35.10–35.14, 35.20–35.28, 35.33, 36.10–36.19, 36.2) and non-invasive (35.00–35.04, 35.96, 36.01–36.07, 36.09) cardiac procedures. It was applied to 5,878 cardiac procedures in UHG members performed between January 1, 1996 and December 31, 2001, and to 1,106 cardiac procedures in Humana members performed between January 1, 2003 and June 30, 2003.

Selected output from the application is provided here as an example of the type of results generated under default settings. Table 2 provides cohort descriptions of health plan members undergoing cardiac procedures from the two health plans. Table 3 details the frequency of SSI indicators among these procedures.

**Table 2:** Characteristics of Health Plan Members Undergoing Cardiac Procedures

	**UnitedHealth Group**	**Humana Inc.**
**Time Period**	1-1-96 to 12-31-01	1-1-03 to 6-30-03
**Cardiac Procedures (N)**	5,787	1,106
**Mean Age at Procedure**	61.2 years	68.7 years
**% Male**	72%	66%
**Mean Chronic Disease Score**	1,708	3,372
**Insurer Plan**		
% Medicare	1,852 (32%)	757 (68%)
% Commercial	3,935 (68%)	349 (32%)
**Hospitals (N)**	104	10

**Table 3:** Surgical Site Infection Indicators Following Cardiac Procedures

	**UnitedHealth Group**	**Humana Inc.**
**Total Procedures**	5,787	1,106
**Any Infection Indicator N (%)**	413 (7.1%)	96 (8.7%)
**Inpatient Infection Indicator **N (%)	153 (2.6%)	73 (6.6%)
Pre-Discharge N (%)	66 (1.1%)	42 (3.8%)
Post-Discharge with Readmission N (%)	90 (1.6%)	51 (4.6%)
**Outpatient Infection Indicator **N (%)	304 (5.3%)	60 (5.4%)
Antibiotic Indicator N (%)	73 (1.3%)	N/A*
Ambulatory Diagnosis N (%)	199 (3.4%)	34 (3.1%)
Emergency Dept Diagnosis N (%)	41 (0.7%)	39 (3.5%)

* Post-procedure antibiotic dispensing data not available

Cardiac procedures among UHG members were performed in 104 hospitals. The number of procedures per hospital ranged from 1 to 273. Overall, 7.1% (413/5,787) of procedures were associated with 457 SSI indicators. The majority of indicators occurred post-discharge in the outpatient setting. Among Humana members, 8.7% (96/1,106) of procedures were associated with SSI indicators. The distribution of infection indicator types was provided as standard output, with selected types shown in Table 2. Although the proportion of cardiac procedures with an inpatient SSI indicator appears to be substantially higher among Humana members compared to UHG members, they are not comparable across the payers since different claims processes result in differing detection rates of claims-based SSI indicators. We focus here on the qualitative ranking of hospitals by SSI indicator rates provided by these algorithms for claims data within a payer system. Prior work has shown that payer data can be successfully combined to yield accurate relative SSI rates among hospitals when adjusting for the payer [16].

Qualitative ranking of hospitals by SSI indicator rates were successfully performed by the provided programs using data from each health plan. We provide additional UHG results as an example of selected output. Among the subgroup of 44 hospitals with claims for ≥ 40 cardiac procedures in UHG members, the percentage of unadjusted SSI indicators ranged from 1.5 to 18.9%. The lowest quartile of hospitals' SSI indicator rates ranged from 1.5–4.4% (median 3.5%), and the highest quartile ranged from 9.8–18.9% (median 12.5%). Inpatient infection indicators ranged from 0% across the entire lowest quartile to 3.7–9.5% (median 5.0%) among the highest quartile, while outpatient infection indicators ranged from 0–3.5% (median 2.8%) in the lowest quartile and 7.1–13.5% (median 9.7%) in the highest quartile. These rates represent SSI indicators among both invasive and non-invasive procedures combined; however, the programs also provide stratification by procedure type.

**Figure 1:**
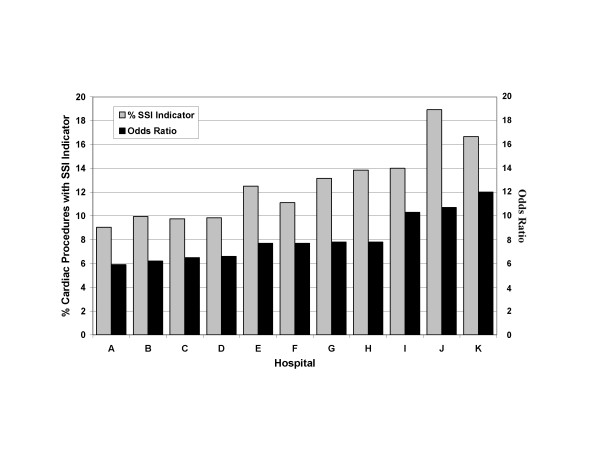
**Claims-based Indicators of Surgical Site Infection**. Graphical display of the hospitals serving UnitedHealth Group members which are in the top quartile of percentage surgical site infection indicators following a cardiac procedure. Hospitals are ranked by their odds ratio compared to a hypothetical control hospital with the median percentage of SSI indicators from among the lowest quartile of hospitals. Odds ratios were derived from a multivariate model controlling for insurer type, sex, chronic disease score, and epoch (see text). Unadjusted percentages of SSI indicators are also shown for each hospital.

When evaluating the multivariate models providing adjusted ranking of hospitals by the percentage of cardiac procedures among UHG members with any SSI indicator, we found that, in this dataset, adjusted and unadjusted results were similar in the highest quartile of SSI rates (Figure 1). Algorithms provided odds ratio estimates for all quartiles in comparison to the 1^st ^quartile (data not shown). In addition, the UHG data showed that Medicare insurer type (OR = 1.4, CI: 1.0, 1.8) and a chronic disease score ≥ 4,500 (OR = 2.0, CI: 1.3, 3.3) were also significantly associated with having an infection indicator within the 30-day period following the procedure. Male gender was not predictive of an SSI indicator.

## Discussion

We previously developed and validated an automated method of using health plan claims data to identify surgical site infections following cardiac procedures [13,16]. We now show that this method can be operationalized for use at the payer-level, without the need for transfer of protected health information to an external analytic center. This capability and provision of a user manual, SAS programs, test datasets, and sample output for public use, enables payers to use this software to routinely and serially assess post-cardiac SSI rates among hospitals frequented by their enrollees. These programs are available without charge [20].

These programs can be applied periodically, e.g., every six or twelve months, to identify hospitals that may warrant closer attention, to assess secular trends, to evaluate the impact of healthcare or health plan polices on SSI indicator rates, or to screen for improvement in specific hospitals previously identified as having potentially high infection rates. After adjusting for health plan, the aggregated data can be combined across plans/payers to provide more precise estimates when a hospital's procedures are spread across several payers, [16] although this capability is not provided in this software version.

The use of SSI indicators based on claims data has several advantages over traditional surveillance. It uses readily available data, requires relatively small investment of resources to maintain after the programs have been configured to operate in a specific claims environment, and is less susceptible to subjective interpretations compared with conventional infection surveillance. In principle, this type of surveillance can be applied to the large majority of institutions that perform cardiac procedures in the United States.

Additionally this approach can identify SSI indicators that occur in the post-discharge setting. It enables identification of SSI indicators resulting in re-hospitalization, even in institutions other than the ones that performed the original procedure; at present, hospitals are rarely able to track these events. It also identifies outpatient infection indicators based upon clinic visits, emergency department visits, and antibiotic prescriptions. The ability to distinguish outpatient from inpatient infection indicators provides further insight into the sources of elevated SSI rates. This is a valuable component of SSI surveillance since post-discharge SSIs are known to constitute the majority of infections, although there is no agreed upon method to identify them.

There are several important caveats in using these programs. First, these indicators are only surrogates for confirmed surgical site infections. Although they have been shown to correlate reasonably well with actual SSI rates at the level of hospitals in prior validation studies, we believe they are most appropriately used at this time to identify hospitals that bear closer scrutiny to determine whether their SSI rates really are high. For that reason, we have configured the programs to focus attention on the hospitals in the top quartile. Although these SSI indicators have been validated as important surrogates for SSI rates,[16] we believe that further validation is needed across additional health plans and that these SSI indicator rates should not be directly equated with actual SSI rates. Second, a substantial amount of the variation between hospital SSI indicator rates (and confirmed SSI rates) is probably caused by variation in case-mix that may not corrected by the factors available in claims data. It will be important to avoid using these rates for inter-hospital comparisons without further accounting for this variation in case-mix. Third, due to the instability of rankings when small numbers are used, these programs suggest that only hospitals performing a minimum number of annual cardiac procedures (default = 100) be assessed. While this threshold number can be changed at the user's discretion, in general, this methodology is unable to meaningfully comment on hospitals performing a very small number of procedures. Decisions on how to adequately assess these hospitals during a time of national standards and public reporting are needed, but beyond the scope of this paper. Finally, these SSI indicator rates are unlikely to be useful in comparing individual surgeons. Problems of case-mix and small sample size are greatly magnified at the level of individual surgeons.

Although these algorithms have been previously validated and are now operationalized for US health payers, they could be modified for use in other countries by finding corresponding claims codes. Nevertheless, given the lack of prior validation in non-US health systems, the selection of corresponding codes and the demonstration that the algorithm applies to non-US medical care would be needed.

## Conclusion

This simplified screening tool based on readily available claims data offers insurers the potential of rapid, periodic, and comprehensive assessments comparing indicators for SSIs across hospitals performing cardiac procedures. Compared to subjective and labor-intensive chart reviews, this methodology applies uniform criteria for SSI indicators and is not limited to the initial hospital stay in which the procedure occurred. Payors can thus identify hospitals with potentially high SSI rates and target further evaluations.

## Availability and Requirements

The programs, test datasets, and user manual are publicly available for downloading at . Programs require SAS version 9.0 (Cary, NC) or above and will run on any SAS platform.

## Authors' contributions

SSH, JML, and RP were responsible for study concept, design, and analysis. JML provided programming expertise. NSBR and SS were responsible for data acquisition and application of the programs. SSH was responsible for drafting of the manuscript, and all authors were responsible for critical revision of the manuscript. Funding was obtained by RP.

## Pre-publication history

The pre-publication history for this paper can be accessed here:


